# Clinical Concepts on Thyroid Emergencies

**DOI:** 10.3389/fendo.2014.00102

**Published:** 2014-07-01

**Authors:** Giampaolo Papi, Salvatore Maria Corsello, Alfredo Pontecorvi

**Affiliations:** ^1^Department of Endocrinology, Catholic University of Rome, Rome, Italy

**Keywords:** hypothyroid coma, thyrotoxic storm, hyperthyroidism, thyrotoxicosis, hypothyroidism, massive goiter

## Abstract

**Objective:** Thyroid-related emergencies are caused by overt dysfunction of the gland which are so severe that require admission to intensive care units (ICU) frequently. Nonetheless, in the ICU setting, it is crucial to differentiate patients with non-thyroidal illness and alterations in thyroid function tests from those with intrinsic thyroid disease. This review presents and discusses the main etiopathogenetical and clinical aspects of hypothyroid coma (HC) and thyrotoxic storm (TS), including therapeutic strategy flow-charts. Furthermore, a special chapter is dedicated to the approach to massive goiter, which represents a surgical thyroid emergency.

**Data Source:** We searched the electronic MEDLINE database on September 2013.

**Data Selection and Data Extraction:** Reviews, original articles, and case reports on “myxedematous coma,” “HC,” “thyroid storm,” “TS,” “massive goiter,” “huge goiter,” “prevalence,” “etiology,” “diagnosis,” “therapy,” and “prognosis” were selected.

**Data Synthesis and Conclusion:** Severe excess or defect of thyroid hormone is rare conditions, which jeopardize the life of patients in most cases. Both HC and TS are triggered by precipitating factors, which occur in patients with severe hypothyroidism or thyrotoxicosis, respectively. The pillars of HC therapy are high-dose l-thyroxine and/or tri-iodothyroinine; i.v. glucocorticoids; treatment of hydro-electrolyte imbalance (mainly, hyponatraemia); treatment of hypothermia; often, endotracheal intubation and assisted mechanic ventilation are needed. Therapy of TS is based on beta-blockers, thyrostatics, and i.v. glucocorticoids; eventually, high-dose of iodide compounds or lithium carbonate may be of benefit. Surgery represents the gold standard treatment in patients with euthyroid massive nodular goiter, although new techniques – e.g., percutaneous laser ablation – are helpful in subjects at high surgical risk or refusing operation.

## Introduction

Emergencies related to thyroid gland diseases are infrequently observed in the clinical practice (1–3). They are caused by either overt dysfunction (4, 5) or marked enlargement of the gland (6) that jeopardize the life of patients, and require admission to intensive care units (ICU) in most cases.

The present paper reviews hypothyroid coma (HC), thyrotoxic storm (TS), and massive goiter, discussing related etiopathogenesis, clinical aspects, diagnostic challenges, and prognosis, and finally presenting therapeutic strategy flow-charts.

## Review Criteria

The content of the present review is based on the previous reviews, original articles, and case reports on thyroid emergencies. We searched the electronic MEDLINE database on September 2013 for a combination of the following search terms: “myxedematous coma,” “hypothyroid coma,” “thyroid storm,” “thyrotoxic storm,” “massive goiter,” “huge goiter,” “prevalence,” “etiology,” “diagnosis,” “therapy,” and “prognosis.” Only papers written in the English language were included in this review.

## Emergency Related to Thyroid Hormone Excess or Deficiency

Either excess or deficiency of thyroid hormones is caused by several thyroid disorders. Whatever is the underlying cause, both thyrotoxicosis and hypothyroidism deeply impact on cardiovascular and digestive systems, basal metabolism, neuro-psychological functions, muscles, and cutis. Rarely, the excess or the defect of thyroid hormone is so severe that jeopardize the life of patients, who should be promptly referred to the emergency department.

Boxes 1 and 2 summarize “classical” definitions of thyrotoxicosis and hypothyroidism and thyroid diseases predisposing to TS and HC, respectively. Really, such definitions easily apply to patients in the ambulatory setting, but do not reflect what commonly happens in critically ill subjects. Yet, it is crucial to establish a correct diagnosis in order to differentiate patients with non-thyroidal illness – i.e., affected by the so-called “euthyroid sick syndrome” (Box 3) – by those truly affected by severe thyroid disorders, as the therapeutic approach changes profoundly.

Box 1 **Definition of thyrotoxicosis. Thyroid diseases causing thyrotoxicosis and predisposing to thyrotoxic storm (TS).**

In this review, “subclinical thyrotoxicosis” was defined as a condition characterized by low-undetectable serum TSH with normal free thyroid hormone concentrations (7). “Overt thyrotoxicosis” was defined as a condition with suppressed serum thyrotropin (TSH) and elevated free thyroid hormone concentrations (8).

A cut-off limit of serum thyroid hormone concentrations precipitating TS cannot be established, as serum free-thyroxine (FT4) and free tri-iodothyronine (FT3) concentrations in thyrotoxic storm (TS) patients are as high as in hyperthyroid ones without TS.

The following thyroid diseases have been described in association with TS:

*Graves’ disease; iodine- and amiodarone-induced thyrotoxicosis; toxic nodular goiter; cytokine- and tyrosine-kinase inhibitors-induced hyperthyroidism; subacute de Quervain’s thyroiditis; post-partum thyroiditis; and radiation thyroiditis*.

*Factitious (voluntary) intake of high-dose l-Thyroxine* has been reported as a cause of TS, too.

Box 2 **Definition of hypothyroidism. Thyroid diseases causing hypothyroidism and predisposing to hypothyroid coma (HC).**

In this review, “subclinical hypothyroidism” was defined as a condition characterized by normal thyroid hormone levels and high serum TSH concentrations (9–11). “Overt hypothyroidism” was defined as a condition with serum TSH concentrations exceeding the normal range associated to low serum free thyroid hormone level (12).

*Chronic (Hashimoto’s) thyroiditis* and *cytokine- and tyrosine-kinase inhibitors-induced hypothyroidism* have been so far reported in association with hypothyroid coma (HC).

*Total thyroidectomy; neck external beam radiotherapy;^131^I treatment of hyperthyroidism; use of anti-thyroid drugs (methimazole, propylthiouracyle), sedatives, and analgesics* have been also reported as a cause of HC.

Box 3 **The euthyroid sick syndrome (ESS).**

Patients affected by critical illness frequently manifest alterations in serum thyroid hormone concentrations, although they do not really suffer from thyroid disease (13). This condition is known as “euthyroid sick syndrome” (ESS), “non-thyroidal illness,” or – less commonly at the moment – “low T3 syndrome” (14).

ESS is the direct consequence of changes in the function of hypothalamus-pituitary-thyroid axis, in thyroid hormone metabolism and even in thyroid gland function *per se*, occurring in critical illness and sometimes induced by drugs (15). So far published studies have reported alterations in deiodinase activity, TSH secretion, hormone binding to serum proteins, thyroid hormone transport into tissues, and the nuclear thyroid hormone receptors.

Starvation and sepsis are the conditions that physician working in the intensive care units (ICU) most commonly deal with and represent the paradigms for ESS (16). The laboratory hallmark of ESS, at least in its early phase, is low serum T3 concentration, typically associated to TSH levels in the low-normal range, so representing a variant of secondary or tertiary hypothyroidism (17). Concurrent low serum T4 concentrations represent a prognostically unfavorable sign (18).

Decreased binding to carrier proteins may occur in ESS (19), supporting the value of free rather than total thyroid hormone measurement in this setting (20). The use of drugs – e.g., furosemide, heparin, non-steroidal anti-inflammatory drugs, amiodarone, anti-epileptic drugs, dopamine, dobutamine, glucocorticoids, and somatostatin – might also have a significant impact on thyroid hormone and TSH levels (21, 22). Thus, TSH measurement alone should be avoided in the context of ICU, as in the absence of thyroid function tests it might be misleading and induce physicians to treat patients for hyperthyroidism.

During the recovery phase from ESS, transient elevation – usually not exceeding 20 mIU/L – in serum TSH concentrations may occur (23), resembling the recovery phase from subacute “de Quervain” thyroiditis. At this point in time, ESS should be accurately differentiated by subclinical hypothyroidism. Serum TSH concentrations above 20 mIU/l, high anti-thyroglobulin and anti-thyroperoxidase autoantibody levels, and/or a diffusely hypoechoic aspect of the thyroid gland on US are in favor of primary hypothyroidism, and should suggest to start levothyroxine substitution therapy. Conversely, studies so far conducted in the literature have demonstrated that levothyroxine substitution therapy is not indicated in patients with ESS (24).

## Hypothyroid Coma

Hypothyroid (or myxedematous) coma (HC) is the result of a very severe, as yet untreated, hypothyroidism (25). It represents an endocrine emergency that should be handled in ICU.

In a background of thyroid hormone deficiency, HC is usually triggered by precipitating factors, as low outside temperatures, systemic (mainly, pulmonary) infections, congestive heart failure (CHF), labor, cerebrovascular events, intake of anesthetics, depressants, neuroleptics, or large liquid amounts (25–31). It manifests rarely, with a prevalence approaching 0.1% of hospitalized hypothyroid patients, and particularly affects female subjects aged >60 years (32). Even if l-thyroxine replacement therapy is quickly and appropriately given, about 15–20% of patients finally die (1).

### Etiology

Most patients referred to the hospital for HC are already followed by the endocrinologist or the general practitioner because of hypothyroidism consequent to autoimmune chronic thyroiditis, thyroidectomy or Graves’ disease treated by radioiodine (^131^I). Usually, these subjects have previously been taking l-thyroxine substitution therapy, unless they have subsequently withdrawn it on their own initiative. Rarely, the cause of HC is not of primary thyroid origin, but is because of reduced TSH excretion by the pituitary gland (e.g., hypopituitarism) (33).

Patients presenting with secondary hypothyroidism have been previously submitted to surgery or radiotherapy because of pituitary adenoma, or are affected by pituitary macroadenoma overwhelming TSH-producing pituitary cells. Some drugs – e.g., amiodarone and lithium – may directly cause hypothyroidism and HC (22–24). Amiodarone is an anti-arrhythmic drug containing high (about 37 mg) iodine amounts. Approximately 15% of patients taking amiodarone develop hypothyroidism or thyrotoxicosis, mainly depending on concurrent thyroid disease (e.g., autoimmune or nodular thyroid disease) and daily iodine intake (21). Amiodarone might cause hypothyroidism inducing inhibition of 5′-deiodinase activity and Wolff–Chaikoff effect. Lithium is commonly used to treat bipolar disorders, inhibits thyroid hormone release from the thyroid gland, and increases thyroid autoimmunity if present before therapy (30, 34, 35). Severe hypothyroidism as a result of impaired iodine uptake by thyrocytes has been described in neoplastic patients treated with tyrosine-kinase selective inhibitors (36). Finally, the case of a Chinese patient referred to the hospital with HC provoked by ingestion of raw bok choy has been reported (37). It has been demonstrated that plants of the family of Brassicaceae contain glucosinolate, a sulfur-containing organic anions bonded to glucose that is hydrolyzed to thiocyanate, a compound known for its competition with iodine. Ingestion of large amounts of Brassicaceae induces goiter and hypothyroidism (38).

### Diagnosis

The diagnosis of HC is based on (1) the history of previous thyroid disease and progressive lethargy; (2) peculiar signs and symptoms (Table 1); (3) serum free T4 (FT4) and free T3 (FT3) concentrations below the normal reference range; and (4) serum TSH concentrations far exceeding the normal reference range. With regard to TSH values, they usually exceed 100 mIU/L in most severe and long-lasting cases; however, in patients with hypopituitarism (where serum TSH concentrations are typically low-normal) (5, 39) and in those with recent-onset hypothyroidism, serum TSH concentrations may not be so high.

**Table 1:** **Symptoms and signs peculiar to hypothyroid coma**.

Coma status
Hypothermia (frequently severe, with body temperature <33°C)
Dyspnea
Generalized edema with yellow and dry cutis
Macroglossia
Bradycardia
Weak wrists
Reduced cardiac sounds
Overweight/obesity
Constipation
Reduced reflexes
Thin and dry hairs
Focal and general seizures (rare)

The diagnosis of HC is relatively simple. However, because serum thyroid hormone and TSH measurement commonly take hours, once arrived at the hospital, besides serum TSH, FT4, and FT3 concentration measurement, patients should undergo further (i.e., first level) examinations:

- *Arterial hemogasanalysis*, to eventually detect hypoxemia, hypercapnia, and respiratory acidosis.
- *Labanalysis*, which may reveal anemia, hyponatraemia, hypoglycemia, high serum creatine kinase, lactate dehydrogenase, transaminases, creatinine, and cholesterol concentrations. Potential mechanisms underlying hyponatraemia in HC patients are represented by increased serum anti-diuretic hormone (ADH) (40) and impaired water excretion and urine output due to reduced delivery of water to the distal nephron (41). The atony of urinary bladder with the consequent urine retention and the high serum creatine kinase concentrations due to rhabdomyolysis may lead to renal failure (15, 42).
- *Electrocardiography*, usually demonstrating sinus bradycardia, low voltages (related to pericardial effusion), Q–T prolongation and flattened or inverted T waves (consequent to myocardial ischemia).
- *Echocardiography*, which may disclose a pericardial effusion associated to cardiomegaly, increased thickness of all cardiac walls and reduced cardiac output.
- Computed tomography (CT) of the brain that is normal in most patients with primary hypothyroidism (although long-lasting hypothyroidism promotes atherogenesis and, then, ischemic encephalopathy), and generally detects pituitary macroadenoma or an empty sella in patients with secondary hypothyroidism.

### Treatment

**Figure 1:**
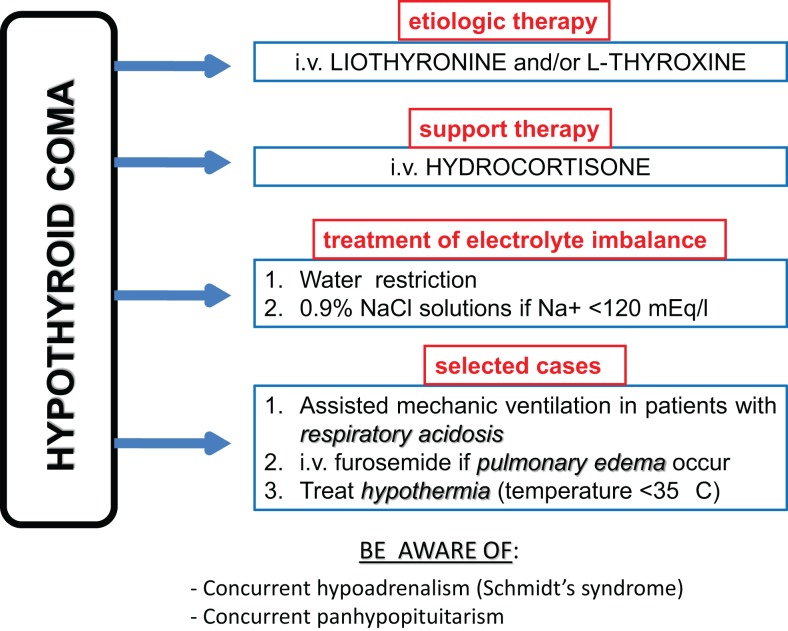
**Flow-chart of therapeutic approach to hypothyroid coma**.

The pillar of HC therapy is l-thyroxine or l-thyroxine plus liothyronine substitution therapy preferably administered intravenously, owing to the poor intestinal absorption related to severe hypothyroidism (Figure 1). Because of the rarity of HC, prospective studies recruiting large series of patients and evaluating the best therapeutic regimens are lacking in the literature. Therefore, this topic is still controversial. The major matter of debate concerns the starting dose of substitution therapy. Some authors (43) recommend starting with high-dose (300–400 mcg/daily) of T4 i.v., but sudden cardiac death has been reported and most recent literature does not support such a regimen. Rapid correction of hypothyroidism may be tolerated by young otherwise fit adults, but these are not the usual patients presenting with HC. Other authors (15, 44) suggest to give liothyronine 10–20 mcg i.v. as bolus initially, followed by 10 mcg every 4 h for the first 24 h and every 6 h for days 2–3, and then to start oral administration if feasible. Alternatively, a T3 plus T4 approach might be used: an initial dose of 250 mcg of intravenous T4 might be administered, followed by 100 mcg 24 h later and 50 mcg per day (i.v. or by mouth) after that. Simultaneously with the first T4 bolus (day 1), a 10 mcg T3 as i.v. bolus is given followed by 10 mcg i.v. every 8–12 h until the patients is conscious and taking maintenance T4 (15).

Beyond thyroid hormone substitution therapy, hydrocortisone 100 mg every 6 h intravenously and treatment of hydro-electrolyte imbalance should be administered. In particular, hyponatraemia should be first corrected through water restriction. 0.9% NaCl solutions (slow at 12 mmol/l/24 h) should be administered to patients with severe hyponatraemia (<120 mEq/L) only. Hypertonic saline should be used cautiously to avoid osmotic demyelinization syndrome (1, 15). The use of diuretics is generally suggested after the administration of hypertonic saline in order to promote a water diuresis (15).

Hypothermia is to be treated through passive and gradual heating (e.g., by blankets); in contrast, the active body heating is not warranted because of the risk of vasodilatation and shock. Endotracheal intubation and assisted mechanic ventilation with constant monitoring of haemogasanalysis parameters are needed in case of severe respiratory acidosis.

At the same time, treatment of precipitating factors, if any, is needed. For instance, large spectrum antibiotics must be administered to patients with pneumonia, after samples for urine and blood cultures have been previously collected awaiting for laboratory report (3). Furthermore, particular attention must be paid to concurrent diseases in the context of autoimmune polyendocrine syndromes. In particular, hypoadrenalism due to Schmidt’s syndrome (association of autoimmune adrenalitis and Hashimoto’s thyroiditis) should be ruled out (45, 46). Nonetheless, hypoadrenalism might occur as the consequence of panhypopituitarism. In any case, until results of hypothalamus–pituitary–adrenal axis study will be available, intravenous glucocorticoids administration is always mandatory together with l-thyroxine therapy, to avoid the occurrence of acute adrenal insufficiency (45). Hypoglycemia is a serious and common complication of HC and should be recognized and treated as soon as possible by intravenous glucose solutions.

Death is caused by respiratory and/or cardiac (arrhythmias, acute myocardial infarction, acute pulmonary edema, and cardiac shock) complications (47, 48). Although both right and left heart failure may occur in HC, the former is a clue to hypothyroidism. Reduced stroke volume should be consequent to either reduced myocardial contractility *per se* or cardiac tamponade. In patients with pulmonary edema, diuretics (particularly, i.v. furosemide) must be added to the standard HC therapy. Torsades de pointes ventricular tachycardia might be the result of prolonged Q–T interval, and therefore patients should be carefully and constantly monitored with particular regard to this life-threatening event. Use of digoxin is not indicated in subjects with tamponade, and should generally be very cautious in HC patients because of prolonged half-life and decreased volume of distribution. If a treatment with digoxin is started, serum digoxin levels should be monitored regularly and dosage should be administered very slowly increasing. Myocardial infarction is not uncommon in subjects with HC and can be precipitated by too high l-thyroxine doses needed to treat HC in patient with underlying ischemic cardiomyopathy. Owing to impaired consciousness, clinical alert related to ischemic events is lacking in HC patients, and therefore cardiac enzymes – also in the absence of typical electrocardiographic features of myocardial infarction – should be routinely measured in such condition.

Importantly, HC patients are at high risk of bleeding caused by an acquired von Willebrand syndrome and reduction in coagulation factors V, VII, VIII, IX, and X (15, 49). Such a risk is reversible with L-T4 substitution therapy (49).

## Thyrotoxic Storm

Thyrotoxic storm represents the extreme consequence of a severe thyrotoxicosis (4, 50). As HC, TS is frequently triggered by typical precipitating factors that occur when patients are already affected by overt (occasionally, subclinical) hyperthyroidism (51).

The main triggering factors so far reported are summarized in Table 2.

**Table 2:** **Known triggers of thyrotoxic storm**.

Withdrawal of anti-thyroid drug therapy
Major surgery (particularly, thyroidectomy)
Iodide compounds intake or radioiodine (^131^I or ^123^I) therapy in patients with Graves disease or autonomously functioning thyroid nodules
Trauma (mainly, in the neck area)
Systemic infections
Pregnancy/parturition
Infection
Diabetic ketoacidosis
Severe emotional stress
Cerebrovascular disease
Pulmonary thromboembolism
Intense exercise
Use of tyrosine-kinase inhibitors
Minor surgery (extraction of teeth)

Fortunately, TS is a rare event mostly affecting hyperthyroid patients who have not been adequately treated or have withdrawn thyrostatic therapy on their own initiative or have undergone surgery (52–54). Its prevalence averages 1% of hospitalized subjects with hyperthyroidism (53) and is more frequent in the female gender, with a female to male ratio of about 3 to 1 (55). In a recent study, an incidence of TS cases in Japan was estimated to be 0.2 persons/100,000 Japanese population/year, accounting for 0.22% of all thyrotoxic patients and 5.4% of thyrotoxic patients admitted to the hospital (55). In the same series, the mean age of TS patients was 45 years, ranging 6–87 years, without differences between men and women (55).

Prognosis is unfavorable in many cases, unless an adequate treatment is quickly done (56). Indeed, adequate therapy reduces TS mortality to 10% cases approximately (56, 57). Death occurs because of multi-organ failure in about 25% of patients and because of CHF in 1 out of 5 cases (55); respiratory failure, arrhythmia, disseminated intravascular coagulation, gastro-intestinal tract perforation, and sepsis represent the cause of death in the remaining cases (55).

### Etiology

Why serum FT4 and FT3 concentrations in TS patients are as high as in hyperthyroid ones without TS, is still a matter of debate. Indeed, the clinical picture characteristic of TS is not related to thyroid hormone levels (55). Nonetheless, patients presenting with TS have a larger amount of catecholamine binding sites ubiquitously than hyperthyroid subjects who do not develop it.

In accordance with the long-standing evidence on the interrelations between thyroid hormone and catecholamines (58), the most agreed etiopathogenetic hypothesis is that, in presence of both a larger availability of adrenergic receptors and a reduction of thyroid hormone binding to TBG (thyroid hormone binding globulin), the leak of catecholamine provoked by an acute event (i.e., triggering factor) finally precipitates TS.

Pregnancy and the post-partum period are both at high risk of TS occurrence (59–61). Indeed, on the one hand, the altered coagulation state peculiar to pregnancy might be worsened by thyrotoxicosis (see Diagnosis section); on the other hand, immune system modulation – and “re-modulation” in the post-natal phase – may give origin to a new-onset autoimmune hyperthyroidism, or reawaken a previously present (i.e., pre-conception) one (59–62).

Recent radioiodine treatment of severely hyperthyroid patients (i.e., subjects manifesting marked signs and symptoms of thyrotoxicosis, suppressed TSH, markedly elevated free T4 and/or free T3, and elevated radioactive iodine uptake) represents a risk factor for the development of TS (63–67). However, it has been demonstrated that it is safe to administer I-131 to patients who are severely hyperthyroid without fear of TS, provided beta blockade drugs are used to control the signs and symptoms (67).

Furthermore, external radiation therapy for neck neoplasm might cause thyroid follicle rupture and, consequently, the leakage of large amounts of pre-formed thyroid hormones into the systemic blood circulation, finally precipitating TS (67). The same mechanism underlies severe thyrotoxicosis and thyrotoxic coma seldom provoked by acute (68) or subacute (69, 70) thyroiditis. In contrast, the etiopathogenesis of the TS occurring in patients with partial hydatiform mole (71) can be ascribed to thyroid hyperfunction with high 24-h RAIU, following beta-HCG follicular cell stimulation.

Anecdotal cases of accidental (72) or voluntary (73) thyroid hormone ingestion unleashing TS have been reported. Finally, analogously to what happens in HC, the use of tyrosine-kinase inhibitors, particularly sorafenib (74), may trigger TS in cancer patients.

### Diagnosis

Clinically, TS presents as multi-organ failure in most cases (75, 76). The organs mainly affected by thyroid hormone excess are the heart – tachyarrhythmia ranging from sinus tachycardia to atrial fibrillation is invariably present (77–79), the nervous system (80, 81), the gastro-intestinal tract (82, 83), and the liver (79, 84). Fever is also a frequent event (85, 86).

History of recent traumas in the neck area should always be searched for in patients without history of thyroid disease (87–90). Occasionally, the clinical picture of apathetic thyrotoxicosis occurs, in particular in older patients (91).

The diagnosis of TS is based on: (1) history of thyroid disease and eventual triggering factors; (2) typical signs and symptoms (Table 3); (3) serum FT4 and FT3 concentrations exceeding the normal reference range and undetectable (<0.1 mIU/L) TSH levels (92).

**Table 3:** **Symptoms and signs peculiar to thyrotoxic storm**.

Fever
Unreasonable anxiety, confusion, delirium up to coma state
Tachyarrhythmia (particularly, atrial fibrillation)
Tachypnea and dyspnea
Congestive heart failure up to cardiac shock
Lerman-means scratch (pleuro-pericardiac sound)
Increased systolic vs. diastolic blood pressure ratio
Hyperhidrosis and skin hyperemia
Generalized tremors
Diarrhea
Nausea
Vomit

Besides altered thyroid function tests, elevation of serum bilirubin levels (seldom associated to jaundice) and transaminase (93), hyperglycemia, low total cholesterol values and electrolyte imbalance (in Asian men, hypokalemia and associated periodic paralysis) are frequently detected. Total serum bilirubin level concentrations >3 mg/dl have been associated to high risk of death in TS patients, independently of jaundice (55).

Nonetheless, an altered coagulation state – in particular, antithrombin deficiency and increased levels of factor VIII – has been observed in patients with TS (94, 95), sometimes inducing disseminated intravascular coagulation (79, 96). Individuals exceptionally presenting with hypoglycemia, associated (97) or not (79) to lactic acidosis, have been described, as well.

The diagnostic criteria for TS were indicated in 1969 by Mazzaferri et al. (98) who included temperature ≥100°F (37.8°C), marked tachycardia, accentuated signs, and symptoms of thyrotoxicosis and evidence of dysfunction in one more of the central nervous, cardiovascular, or gastro-intestinal systems.

Burch and Wartofsky (56) introduced a punctual score system to identify TS patients definitely. Such score system was composed of the following items: temperature (ranging from 37.2 to >40°C); central nervous system effects (from mild agitation to coma, passing through the psychotic state); gastro-intestinal-hepatic dysfunction (including diarrhea, nausea/vomiting, abdominal pain, and unexplained jaundice); heart rate (ranging 90 to ≥140 bpm); atrial fibrillation; heart failure (low score pedal edema – high score pulmonary edema); and negative or positive precipitant history. A score of 45 or more is highly suggestive of TS; a score of 25–44 supports the diagnosis; a score below 25 makes TS unlikely.

Recently, Akamizu et al. (55) have reported the diagnostic criteria of TS based on Japanese Nationwide surveys. They utilized five symptoms – i.e., central nervous system manifestations, fever (≥38°C), tachycardia (≥130 bpm), CHF, and gastrointestinal (GI)-hepatic manifestations – to define diagnostic criteria for “definite” (TS1) or “suspected” (TS2) cases of TS. Based on the combination of thyrotoxicosis and the above mentioned symptoms, they defined TS1 patients as those presenting with thyrotoxicosis and at least three combinations of fever, tachycardia, CHF, or GI/hepatic manifestations. Suspicion for TS (TS2 cases) should raise when patients meet the diagnostic criteria for TS1, except that FT3 or FT4 values are not available but data before or after the episode suggest that they are thyrotoxic at the time of TS.

Differences in TS diagnostic criteria between Burch and Wartofsky (56) and Akamizu et al (55) are reported in Table 4. Actually, the score system for TS proposed by Akamizu neither significantly differs from, nor adds substantial advantages to, the prior Burch–Wartofsky criteria. Nevertheless, it should be highlighted that Japanese population enrolled in Akamizu et al.’s study is different from American and European populations by the genetic point of view. One of possible examples of such a difference is that, in their series, TS patients affected by Graves’ disease exceeded 95%, whereas in non-Asian series – particularly in those from Europe, where areas of mild to moderate iodine insufficiency are still present – a higher incidence of patients with toxic nodular goiter, iodine-induced hyperthyroidism, or destructive thyroiditis should be expected. Therefore, certain clinical manifestations, like as jaundice, may be expressed at different degrees in Asian compared to non-Asian patients; analogously, a difference in patient’s response to treatment should be taken into account, too.

**Table 4:** **Differences in diagnostic criteria for thyrotoxic storm between Burch and Wartofsky and the Japanese survey**.

Criterion	Burch and	Akamizu et al.
	Wartofsky	
Thyrotoxicosis	Not included	Pre-requisite
Scoring system	Included	Not included
Fever	≥37.2°	≥38°C
Heart rate	≥90 bpm	≥130 bpm
Atrial fibrillation	Included	Not included
Heart failure	Pedal edema to pulmonary edema	NYHA classification class IV or Killip classification ≥III
Serum bilirubin concentrations	Not included	>3 mg/dL
Jaundice	Included	Not included

### Treatment

**Figure 2:**
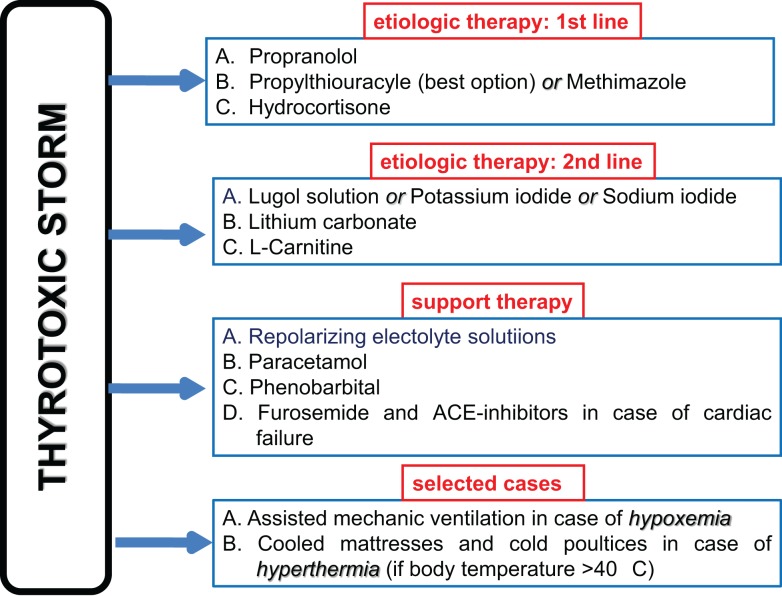
**Specific and support therapy in patients with thyroid storm**.

The initial treatment of TS is resuscitation. Patients are at high risk of severe hypoxemia and tissue ischemia and need oxygen (Figure 2). Most of them need intubation and mechanic ventilation. Furthermore, it is mandatory to start fluid infusion and electrolyte correction as soon as possible.

Specific TS treatment – i.e., therapy given to reduce thyroid hormone excess in the bloodstream and its deleterious peripheral effects – relies on the following medications, in order of importance (99–101):

1. *Beta-blockers*: propranolol 1–2 mg intravenously or 40–80 mg *per os* every 8 h is the drug of choice because, on the one hand, contrasts the increased binding of catecholamine to beta-adrenergic receptors, on the other hand, reduces the T4 to T3 peripheral deiodination.
2. *Thyrostatics*: methimazole 15–20 mg every 6 h or propylthyouracyle with a loading dose of 500–1000 mg followed by 250 mg every 4 h (15, 102). The latter should be preferred because it reduces the T4 to T3 peripheral deiodination. However, methimazole may be given intravenously in case the patient is not able to swallow and a total parenteral nutrition is needed. It should be mentioned that rectal administration of both methimazole and propylthyouracyle is allowed, at a dose of 400–600 mg every 6 h and 20–40 mg every 8–6 h, respectively.
3. *Large iodine amount*: Lugol solution (10 drops three times daily) or saturated potassium iodide solution (5 drops three times daily) *per os* or sodium iodide 500–1000 mg daily intravenously inhibits thyroid hormone leakage by the thyroid gland. Iodine should be administered not sooner than 1 h after anti-thyroid drug administration.
4. *Glucocorticoids*: hydrocortisone 100 mg intravenously every 6–8 h reduces the T4 to T3 peripheral deiodination.
5. *Lithium carbonate*: 300 mg every 6–8 h inhibits proteolysis of colloid, and is administered as an alternative to inorganic iodine to limit the release of pre-formed thyroid hormone into the bloodstream.

*Extracorporeal plasmapheresis* is an additional tool for removing circulating thyroxine in patients who do not respond quickly to conventional standard therapy (103). Besides, a support therapy including liquids (particularly, repolarizing electrolyte solutions), antipyretics (e.g., paracetamol) and phenobarbital (which plays a sedative role and reduces serum FT4 and FT3 concentrations by increasing thyroid hormone metabolism) may be of benefit. Because salicylates compete with thyroid hormone for binding to carrier proteins and may cause a transient increase in serum free T4 concentrations, their use should be avoided in the treatment of TS. *l-*Carnitine, a naturally occurring inhibitor of thyroid hormone nuclear uptake, was able to reverse and prevent symptoms of hyperthyroidism in a randomized, double-blind, and placebo-controlled clinical trial (104), and has been effectively used in successive thyroid storms in association with low doses of methimazole (105).

If CHF is present, not only liquid should be administered cautiously, but large amounts of diuretics (intravenous furosemide) are frequently required in association with specific cardiac support therapy (78). Indeed, cardiac insufficiency in TS patients is mostly categorized as NYHA functional class IV and/or Killip class III or IV, and many patients manifest pulmonary edema and high output-induced cardiogenic shock (55). ACE-inhibitors in association with beta-blockers are the cornerstones of therapeutic strategy in TS patients with heart failure, as much as heart rate (≥130 bpm) tachycardia occurs.

Atrial fibrillation is observed in up to 40% of TS patients (55), in whom heparin therapy is obviously indicated; however, changes occurring in patients with TS may lead to heparin resistance and pro-coagulation state (94–96).

High body temperatures must be reduced by cooled mattresses and cold poultices. Moreover, in the presence of infections, soon after blood and urine specimens have been obtained for cultures, large spectrum antibiotics should be promptly started waiting for culture results.

In the TS Japanese series, factors relevant to the mortality were presence of multi-organ failure; presence of disseminated intravascular coagulation; and presence of shock. In the same series, Glasgow Coma Scale and blood urea nitrogen values predicted irreversible damages (55). Thus, these conditions should be recognized and adequately treated.

Once the patient is stable, the differential diagnosis of thyroid disease underlying TS should be accurately investigated, with the aim of distinguishing thyroid hyperfunction, destructive thyroiditis or thyrotoxicosis factitia. Based on the patient’s history and clinical presentation, the work-up should consider the use of thyroid ultrasound, possibly including color-power imaging for evaluation of gland’s volume, echogenicity, vascularity, and presence of nodular disease. Serum anti-TSH receptor autoantibody concentrations should be measured in cases suspected, and without previous history, of Graves’ disease. If destructive thyroiditis or thyrotoxicosis factitia is suspected, serum thyroglobulin measurement and, in the latter, measurement of fecal thyroid hormone excretion should be obtained; nonetheless, in both cases Tc99m thyroid scan should detect scanty/absent radionuclide uptake in the thyroid bed.

## Compressive Symptoms Caused by Huge Goiter and Aggressive Thyroid Tumors

Nowadays, patients affected by goiter – diffuse or nodular – are rarely submitted to emergency thyroidectomy or tracheotomy due to compressive symptoms on esophagus and/or trachea, such as dysphagia, dysphonia, and dyspnea. Indeed, appropriate therapeutic strategies (e.g., surgery or radioiodine therapy) are usually applied before massive goiter development.

The disease mainly affects female gender and the prognosis is usually favorable, except for malignancies (106, 107).

### Etiology

Thyroid disorders eventually causing emergency due to goiter mass-effect are the following: long-lasting benign huge goiters, for which patients previously refused surgery or radioiodine therapy (6, 108–110); massive hematoma occurring within a thyroid nodule (111, 112); primary malignancies of the thyroid gland (particularly, anaplastic carcinoma) (113); metastases to thyroid (114); and fibrous Riedel’s thyroiditis (115).

### Diagnosis and therapy

The diagnosis of goiter-related compressive symptoms is based on symptoms and signs complained by patients. Imaging examinations either confirm the clinical suspicion or show the surgeon goiter’s extension toward the neighboring structures, and particularly cleavage plans. The clinical picture is characterized by dyspnea with hypoxemia and respiratory acidosis, stridor, dysphonia, dysphagia, and difficult or impossible extension/flexion of the neck (6, 108–110). Rarely, superior vena cava syndrome (116, 117) or even chylothorax (118) occur. Huge mediastinal goiter causing precordial pain has been reported in one patient (119).

Symptoms of compression of the tracheal tree might be relieved by the administration of a CPAP mask. This notwithstanding, surgery represents the gold standard treatment in patients with massive goiter (120, 121). Ultrasound, CT and magnetic resonance (MRI) of the neck represent useful diagnostic tools before surgery (122, 123). Some ultrasound aspects (hypoechogenicity, punctate calcifications, undefined margins, high intranodal vascularity, and more tall than width lesion) are peculiar to thyroid malignant nodules (122), and suggest the need of performing fine needle aspiration biopsy. Indeed, a pre-surgical diagnosis of thyroid malignancy obtained by cytological examination should induce the surgeon to perform a total rather than a decompressive only, partial thyroidectomy. On the contrary, in case of benign goiter, surgery should be more limited (e.g., sub-total or near-total thyroidectomy), to reduce the risk of damaging one or both recurrent laryngeal nerves and/or causing permanent hypoparathyroidism.

^99m^Tc or radioiodine (^123^I or ^131^I) thyroid scan is indicated to detect hot nodules and intrathoracic goiter (109, 123). Indeed, in patients who present huge toxic multinodular goiter and are at high surgical risk and/or refuse operation, radioiodine (^131^I) therapy should obtain good results on compressive symptoms (109, 124). Recently, radioiodine therapy and percutaneous laser ablation have been proposed as helpful alternative methods to effectively reduce goiter size in subjects affected by euthyroid nodular goiter containing “cold” nodules on thyroid scan (125–127).

In patients affected by Riedel’s thyroiditis, a rare form of fibrosing thyroiditis, when therapy with glucocorticoids fails to improve the clinical picture and symptoms are so severe that surgery is mandatory, isthmus resection is usually enough to relieve compressive complaints (115).

Urgent tracheotomy is performed in cases of anaplastic thyroid carcinoma (113), poorly differentiated thyroid carcinoma or thyroid metastases (114), when the neoplasm is not confined to the neck or invades surrounding tissues so widely that cleavage plans are undetectable, but palliative surgery is mandatory due to acute respiratory insufficiency. Patients with massive goiter have an extremely high risk for local complications when submitted to tracheotomy: therefore, if any, this procedure should be performed in an operating room by skilled surgeons.

## Conflict of Interest Statement

The authors declare that the research was conducted in the absence of any commercial or financial relationships that could be construed as a potential conflict of interest.

## Appendix

### Key points

- Severe excess or defect of thyroid hormone represent rare conditions jeopardizing the life of patients in most cases.
- It is crucial to differentiate patients with non-thyroidal illness – i.e., affected by the so-called “euthyroid sick syndrome” – by those truly affected by severe thyroid disorders, as the therapeutic approach changes profoundly.
- Both HC and TS are triggered by peculiar *precipitating factors*, which occur in patients with severe hypothyroidism or thyrotoxicosis, respectively.
- The pillars of HC’s therapy are high-dose l-thyroxine and/or tri-iodothyroinine; i.v. glucocorticoids; treatment of hydro-electrolyte imbalance (mainly, hyponatraemia); treatment of hypothermia; often, endotracheal intubation and assisted mechanic ventilation are needed.
- Therapy of thyroid storm is based on beta-blockers, thyrostatics, and i.v. glucocorticoids; eventually, high-dose of iodide compounds or lithium carbonate may be of benefit.
- Surgery represents the gold standard treatment in patients with euthyroid massive nodular goiter, although new techniques – e.g., percutaneous laser ablation – are helpful in subjects at high surgical risk or refusing operation.
